# The Energy Values of Food

**Published:** 1921-09-24

**Authors:** 


### The Energy Values of Food.

The ordinary citizen who consumes a mixed diet troubles very little, if at all, about the constituents of each meal, and probably does all the better by following taste rather than science. When large numbers of individuals, however, are rationed in such a way that choice cannot enter into their nutrition, big blunders may be made by those in authority who dictate the menu. Hence the need of exact knowledge of the properties of all the ordinary articles of food. We have received from the War Office a copy of a book, which is already a classic in its own sphere, entitled "Analyses and Energy Values of Foods,"* by Captain R. H. A. Plimmer, D.Sc., who is attached to the Directorate of Hygiene at the War Office and is Head of the Biochemical Department, Rowett Research Institute of Animal Nutrition, at the University of Aberdeen. Up to the present time no work of the kind has been attempted in this country on such an extensive scale, and there has been no standard of English analytical figures. Large institutions of every description, prison establishments, research expeditions, and all departments of military and civil hygiene experience from time to time the need for scientific insight into the nutritive value of prescribed diets, and the volume before us, which is published at moderate cost, embraces an astonishing variety of data, the result of exact experimentation. The analyses number about 900 and the number of weighings involved was over 20,000. Each analysis, so far as material was available, was carried out in duplicate, and the mean figure is given. The proportion of water, ash, sodium chlorate, protein, carbohydrate, and fat have been estimated in various kinds of meat, poultry, and game; in milk, eggs, cheese, butter, and other fats; in fish, vegetables, cereals, fruits, nuts, and other foods. An interesting feature is the determination of acidity. The energy value has been calculated from the analyses of protein, carbohydrate, fat by multiplication with the usual physiological factors—namely, 4.1, 4.1, 9.3 respectively. The author suggests deducting about one-tenth from the total if digestibility is to be taken into consideration. A summary of analyses adds much to the general usefulness of the book, and in a highly interesting but too brief appendix the accessory food factors or vitamines are discussed and their presence in certain fresh foods is indicated. These valuable conclusions have been adapted from the report of the Medical Research Committee in 1919. It is noteworthy, in view of the oft-repeated fallacy that "foreign meat contains no nourishment," that some of the meat used for analysis consisted of frozen Argentine beef and de-frosted New Zealand mutton.

* *Analyses and Energy Values of Foods*, by R. H. A. Plimmer, D.Sc., Captain attached to Directorate of Hygiene, War Office. Published by H.M. Stationery Office, Kingsway, W.C. 2. Price 6s. net.

